# Supplementary material for: Effect of Upregulation of Transcription Factor TFDP1 Binding Promoter Activity Due to RBP4 g.36491960G>C Mutation on the Proliferation of Goat Granulosa Cells
Source: Cells. 2022 Jul 8;11(14):2148. doi: 10.3390/cells11142148 (PMC9321149; doi:10.3390/cells11142148)
Supplement: Supplementary file 1 [file cells-11-02148-s001.zip › cells-1780863-supplementary - PUB/Table S1.pdf]

Table S1 PCR reaction procedure of SNP-KASP

Temperature (℃)	Time (second)	Cycle
94℃	15 min	Hot-start activation
94℃	20 s	10 cycles
61-55℃	60 s	
94℃	20 s	26 cycles
55℃	60 s	
