# Supplementary material for: Effect of Upregulation of Transcription Factor TFDP1 Binding Promoter Activity Due to RBP4 g.36491960G>C Mutation on the Proliferation of Goat Granulosa Cells
Source: Cells. 2022 Jul 8;11(14):2148. doi: 10.3390/cells11142148 (PMC9321149; doi:10.3390/cells11142148)
Supplement: Supplementary file 1 [file cells-11-02148-s001.zip › cells-1780863-supplementary - PUB/Table S2.pdf]

Table S2 Reaction system of SNP-KASP

KASP typing reaction system (DNA diluted to final concentration of 5-50ng per reaction)				
Components	Wet DNA method (μL)		Dry DNA method (μL)	
DNA*	2.5	5	-	-
2xMaster Mix	2.5	5	2.5	5
Primer mix	0.07	0.14	0.07	0.14
