# Supplementary figures and images for: Effect of Upregulation of Transcription Factor TFDP1 Binding Promoter Activity Due to RBP4 g.36491960G>C Mutation on the Proliferation of Goat Granulosa Cells
Source: Cells. 2022 Jul 8;11(14):2148. doi: 10.3390/cells11142148 (PMC9321149; doi:10.3390/cells11142148)

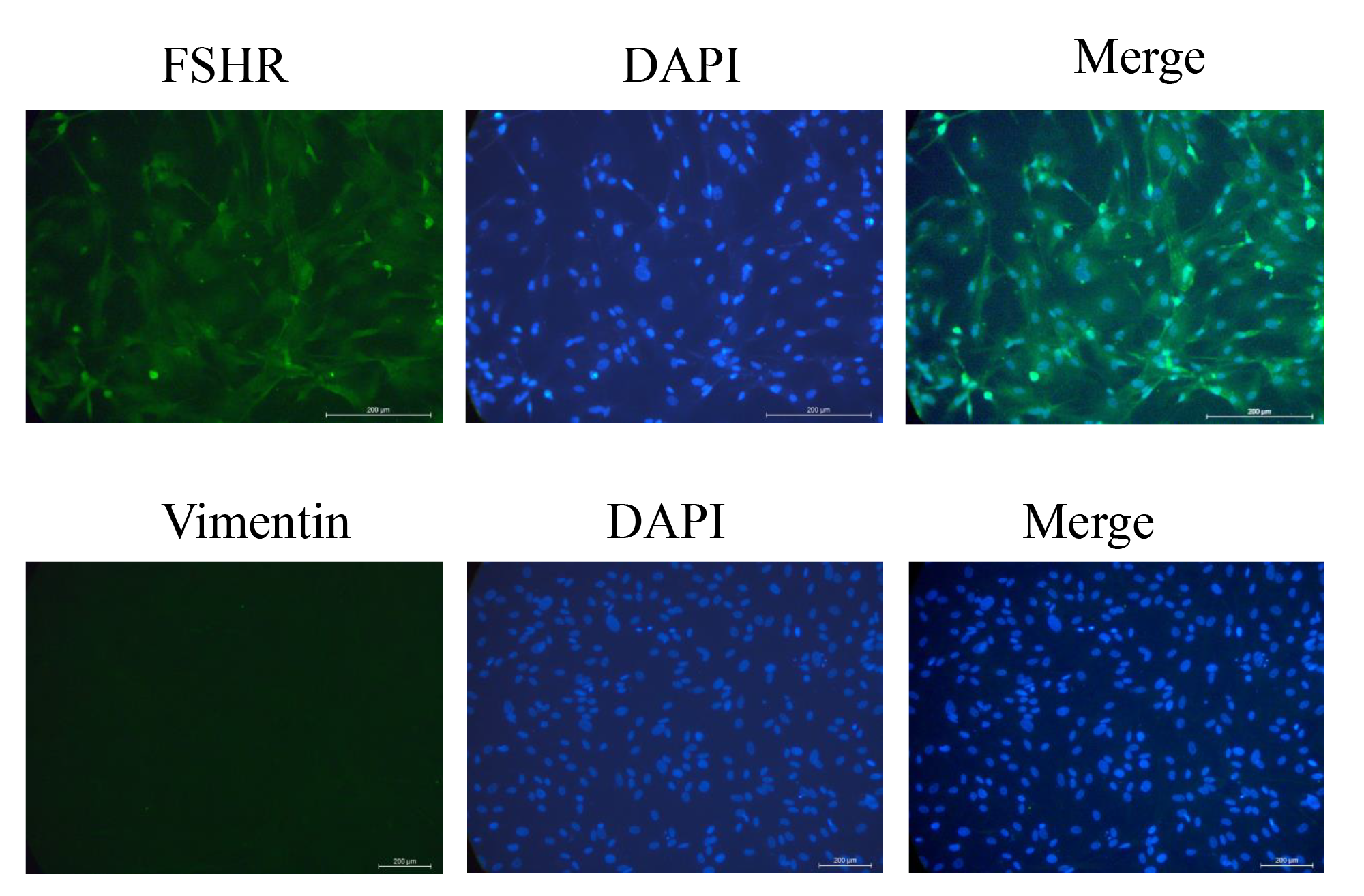

Supplement: Supplementary file 1 [file cells-11-02148-s001.zip › cells-1780863-supplementary - PUB/Figure S1.tif]

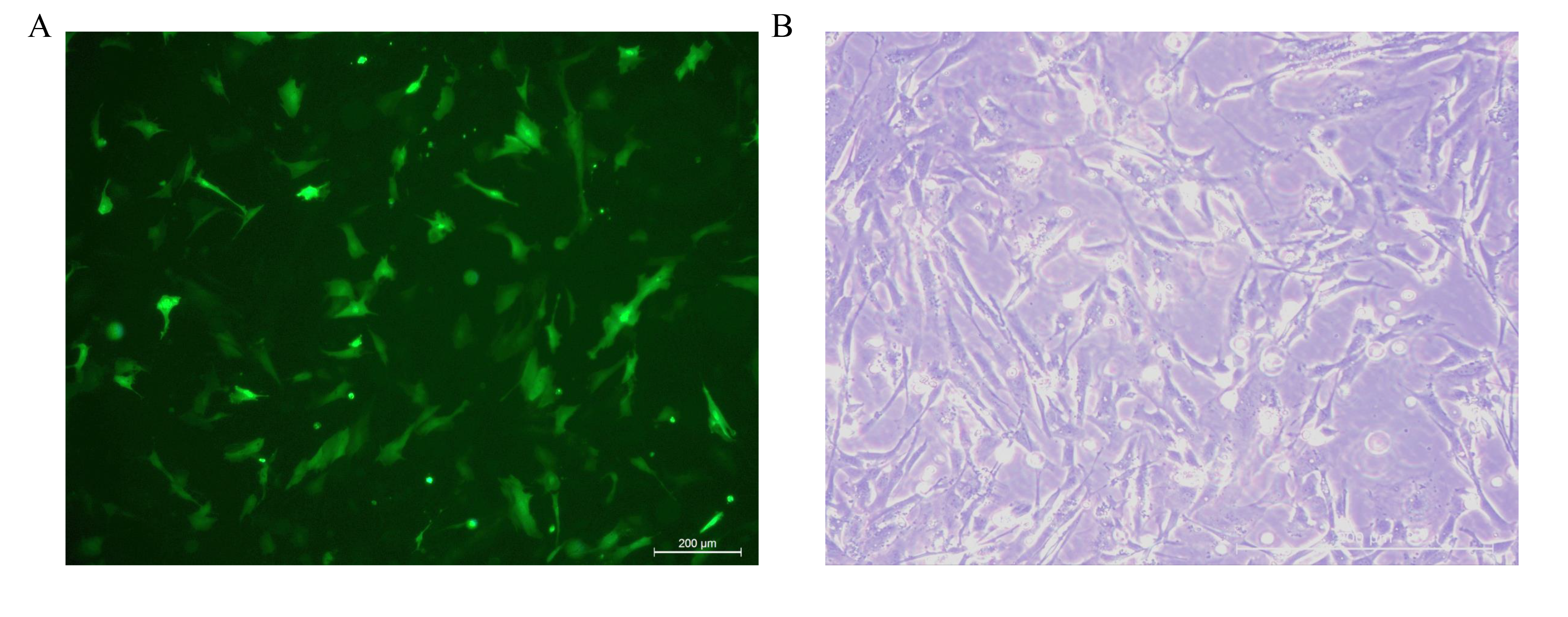

Supplement: Supplementary file 1 [file cells-11-02148-s001.zip › cells-1780863-supplementary - PUB/Figure S2.tif]
